# Supplementary material for: Reconciling Mining with the Conservation of Cave Biodiversity: A Quantitative Baseline to Help Establish Conservation Priorities
Source: PLoS One. 2016 Dec 20;11(12):e0168348. doi: 10.1371/journal.pone.0168348 (PMC5173368; doi:10.1371/journal.pone.0168348)
Supplement: S1 Dataset — (ZIP) [file pone.0168348.s002.zip › Taxa/Serra Norte/SN_2013/N5S/Lista N5S-08.pdf]

CAVIDADE N5S-0008

Classe	Ordem	Fam/Outros	Gên/Outros	Espécie	S-U	U-S	Zon.
Annelida	Oligochaeta			jovem	2		E
Arachnida	Acari	Eupodidae		sp.1	X		P/A
Arachnida	Acari	Laelapidae		sp.3	X		A
Arachnida	Acari	Macronyssidae		sp.1	X		P
Arachnida	Acari	Mesostigmata		jovem	X		E/A
Arachnida	Acari	Prostigmata		jovem		X	
Arachnida	Acari	Rhagidiidae		sp.5	X		A
Arachnida	Acari	Rhagidiidae		sp.7	X		A
Arachnida	Acari	Trombidiformes		sp.49	X		P
Arachnida	Acari	Trombidiidae		sp.15	X		P/A
Arachnida	Amblypygi	Charinidae	*Charinus*	sp.1	7		E/P/A
Arachnida	Amblypygi	Phrynidae	*Heterophrynus*	*longicornis*		X	
Arachnida	Araneae	Caponiidae		sp.1	X	X	P/A
Arachnida	Araneae	Ctenidae		jovem		X	
Arachnida	Araneae	Drymusidae	*Drymusa*	*spelunca*	X	X	E
Arachnida	Araneae	Filistatidae		sp.1	X		E
Arachnida	Araneae	Mysmenidae	*Microdipoena*	sp.1	X		E
Arachnida	Araneae	Ochyroceratidae	*Ochyrocera*	sp.1	X		P/A
Arachnida	Araneae	Pholcidae	*Leptopholcus*	sp.1	X		E
Arachnida	Araneae	Pholcidae	*Mesabolivar*	sp.1	X		E
Arachnida	Araneae	Salticidae		sp.1	X		E
Arachnida	Araneae	Salticidae		sp.2	X		E
Arachnida	Araneae	Salticidae		sp.9	X		E
Arachnida	Araneae	Salticidae		sp.11	X		A
Arachnida	Araneae	Scytodidae	*Scytodes*	sp.1	3		E
Arachnida	Araneae	Segestriidae		jovem	X		E
Arachnida	Araneae	Theraphosidae		jovem	1		E
Arachnida	Araneae	Theridiidae	*Theridion*	sp.1	X		E/P
Arachnida	Araneae	Theridiosomatidae	*Plato*	sp.1	X	X	E/A
Arachnida	Opiliones	Escadabiidae		sp.1	X	X	P/A
Arachnida	Opiliones	Gonyleptidae	*Spinopilar*	sp.1	X		
Arachnida	Opiliones	Stygnidae	*Protimesius*	*gracilis*	1	X	E
Arachnida	Pseudoscorpiones	Bochicidae		sp.1	X		P/A
Arachnida	Pseudoscorpiones	Chthoniidae	*Pseudochthonius*	sp.1	X		P
Arachnida	Pseudoscorpiones	Chthoniidae	*Pseudochthonius*	sp.2		X	E
Chilopoda	Geophilomorpha	Ballophilidae	*Ityphilus*	sp.1	1		E
Chilopoda	Geophilomorpha	Geophilidae		sp.3	1		A
Chilopoda	Geophilomorpha			jovem		X	
Chilopoda	Scolopendromorpha	Cryptopidae	*Cryptops*	jovem	1		E/A
Chilopoda	Scolopendromorpha	Scolopocryptopidae	*Tidops*	jovem	1		E
Chilopoda	Scutigeromorpha	Pselliodidae	*Sphendononema*	*guildingii*	4		P/A
Diplopoda	Polydesmida	Chelodesmidae		sp.3	1		E
Diplopoda	Polydesmida	Pyrgodesmidae		sp.1	X	X	A
Diplopoda	Spirostreptida	Pseudonannolenidae	*Pseudonannolene*	sp.1		X	
Entognatha	Collembola	Cyphoderidae		sp.1	X		P
Entognatha	Collembola	Entomobryidae		sp.9	X		E
Entognatha	Collembola	Paronellidae		sp.1	X		E
Entognatha	Collembola	Paronellidae		sp.4	X		E/P/A
Entognatha	Collembola	Sminthuroidea		sp.2	X		A

Entognatha	Collembola			sp.		X	
Entognatha	Diplura	Campodeidae		sp.1	X	X	E
Insecta	Archaeognatha	Meinertellidae		sp.1		X	
Insecta	Blattodea			jovem	1	X	E/P
Insecta	Coleoptera	Carabidae	*Coarazuphium*	sp.n.2		X	
Insecta	Coleoptera	Carabidae		sp.		X	
Insecta	Coleoptera	Carabidae		sp.3	X		P
Insecta	Coleoptera	Carabidae		sp.12	X		E
Insecta	Coleoptera	Chrysomelidae		sp.3	X		E
Insecta	Coleoptera	Chrysomelidae		sp.4	X		E/P
Insecta	Coleoptera	Chrysomelidae		sp.6	X		E
Insecta	Coleoptera	Dytiscidae		sp.12A	X		E
Insecta	Coleoptera	Scydmaenidae		sp.3	X	X	P/A
Insecta	Coleoptera	Staphylinidae	Brachyglutini	sp.1		X	
Insecta	Coleoptera	Staphylinidae	Pselaphinae	sp.3	X	X	P
Insecta	Coleoptera	Staphylinidae	Pselaphinae	sp.12		X	
Insecta	Coleoptera	Tenebrionidae	Alleculinae	jovem		X	
Insecta	Diptera	Phoridae	Metopininae	sp.	X		P/A
Insecta	Diptera	Phoridae	Phorinae	sp.	X		A
Insecta	Diptera	Psychodidae	*Sciopemyia*	*sordellii*	X	X	
Insecta	Diptera	Sciaridae		sp.	X		P
Insecta	Heteroptera	Dipsocoroidea		sp.	X		P/A
Insecta	Heteroptera	Reduviidae	Reduviinae	sp.	1		E
Insecta	Heteroptera	Veliidae	*Paravelia*	jovem	X		E
Insecta	Homoptera	Cixiidae		jovem	X	X	E/P/A
Insecta	Hymenoptera	Braconidae		sp.3	X		E
Insecta	Hymenoptera	Chalcidoidea		sp.2	X		E
Insecta	Hymenoptera	Formicidae	*Cephalotes*	sp.1	X		E
Insecta	Hymenoptera	Formicidae	*Eurhopalothrix*	sp.1	X		A
Insecta	Hymenoptera	Formicidae	*Gnamptogenys*	sp.1	X		E
Insecta	Hymenoptera	Formicidae	*Gnamptogenys*	sp.2	X		A
Insecta	Hymenoptera	Formicidae	*Hypoponera*	sp.		X	
Insecta	Hymenoptera	Formicidae	*Hypoponera*	sp.1	X		E/P/A
Insecta	Hymenoptera	Formicidae	*Hypoponera*	sp.2	X		E/P/A
Insecta	Hymenoptera	Formicidae	*Odontomachus*	sp.1	X		E
Insecta	Hymenoptera	Formicidae	*Pachycondyla*	sp.2	X		E/P/A
Insecta	Hymenoptera	Formicidae	*Paratrechina*	sp.2	X		E
Insecta	Hymenoptera	Formicidae	*Pheidole*	sp.1	X		E
Insecta	Isoptera	Termitidae	*Nasutitermes*	sp.	X		E/P
Insecta	Lepidoptera	Tineoidea		sp.1	X		E
Insecta	Lepidoptera	Tineoidea		sp.2	X		E
Insecta	Lepidoptera	Tineoidea		sp.3	X		P
Insecta	Lepidoptera			jovem		X	
Insecta	Orthoptera	Phalangopsidae	*Paraclodes*	sp.	37	X	E
Insecta	Orthoptera	Phalangopsidae	*Phalangopsis*	sp.	2	X	
Insecta	Psocoptera	Epipsocidae	*Epipsocus*	sp.1	X		E
Insecta	Psocoptera	Epipsocidae	*Epipsocus*	sp.2	X		E
Insecta	Psocoptera	Psyllipsocidae		jovem	X		E
Insecta	Siphonaptera	Rhopalopsyllidae		sp.1	X		A
Insecta	Thysanura	Nicoletiidae		sp.1	X		E
Gastropoda	Pulmonata	Systrophiidae	*Happia*	sp.	X	X	E/P/A
Malacostraca	Isopoda	Armadillidae		sp.1	X		E

Malacostraca	Isopoda	Philosciidae		sp.2	X		E
Malacostraca	Isopoda	Scleropactidae	*Circoniscus*	*buckupi*	X		A
Malacostraca	Isopoda	Scleropactidae		sp.		X	
Symphyla		Scutigerellidae		sp.	X	X	E/P/A
Amphibia	Anura	Strabomantidae	*Pristimantis*	cf. *fenestratus*	5		E/P
Mammalia	Chiroptera	Emballonuridae	*Peropteryx*	*kappleri*	10		E/P/A
Mammalia	Chiroptera	Furipteridae	*Furipterus*	*horrens*	5		P/A
